# Direct Wideband Coherent Localization by Distributed Antenna Arrays

**DOI:** 10.3390/s19204582

**Published:** 2019-10-21

**Authors:** Nenad Vukmirović, Miljko Erić, Miloš Janjić, Petar M. Djurić

**Affiliations:** 1 School of Electrical Engineering, University of Belgrade, 11000 Belgrade, Serbia; nenad.vukmirovic@ic.etf.bg.ac.rs (N.V.); milos.janjic@ic.etf.bg.ac.rs (M.J.); 2 Innovation Center of the School of Electrical Engineering, University of Belgrade, 11000 Belgrade, Serbia; 3 Department of Electrical and Computer Engineering, Stony Brook University, New York, NY 11794, USA; petar.djuric@stonybrook.edu

**Keywords:** wideband direct position estimation, distributed antenna array, spatial coherence, phase of arrival, indoor localization, 5G, mmWave, massive MIMO

## Abstract

We address wideband direct coherent localization of a radio transmitter by a distributed antenna array in a multipath scenario with spatially-coherent line-of-sight (LoS) signal components. Such a signal scenario is realistic in small cells, especially indoors in the mmWave range. The system model considers collocated time and phase synchronized receiving front-ends with antennas distributed in 3D space at known locations connected to the front-ends via calibrated coaxial cables or analog radio frequency over fiber links. The signal model assumes spherical wavefronts. We propose two ML-type algorithms (for known and unknown transmitter waveforms) and a subspace-based SCM-MUSIC algorithm for wideband direct coherent position estimation. We demonstrate the performance of the methods by Monte Carlo simulations. The results show that even in multipath environments, it is possible to achieve localization accuracy that is much better (by two to three orders of magnitude) than the carrier wavelength. They also suggest that the methods that do not exploit knowledge of the waveform have mean-squared errors approaching the Cramér–Rao bound.

## 1. Introduction

Localization in cellular systems has been attracting much interest in the research community for many years. The key challenge of localization is to improve the accuracy from hundreds of meters in the second generation (2G) (a list of abbreviations can be found at the end of the paper) to 1cm or better in 5G [1]. If one can accomplish this in indoor environments, location-based services (LBS) will be improved considerably. Localization can also be used for LAC (location-aided communication), where one takes advantage of the information about terminal locations in different ways to improve the communication links [2]. This paper focuses on methods that achieve accuracy much better than the carrier wavelength, which can be used both for LAC and LBS.

The classical two-step localization methods, based on measuring received signal strength (RSS), time of arrival (ToA), time difference of arrival (TDoA), and angle of arrival (AoA), as well as the one-step method called direct position determination (DPD) [3,4] provide root-mean-squared errors (RMSE) that are greater than the carrier wavelength. The reason for this relatively high inaccuracy is that these methods are adapted to environments/systems where phase differences between distant antennas either carry no transmitter location information (the full spatial coherence across the entire distributed receiving system is not assumed) or that this information is simply ignored.

With spatial coherence, the accuracy of localization can be much improved. The authors in [5,6] analyzed the accuracy gains of coherent localization in passive systems and multiple-input multiple-output (MIMO) radars (active localization), respectively. Unlike [6], in which the receiving system was synchronized to the transmitters that are at known locations (radar/active localization), in our paper, the receiving system is not synchronized to a transmitter, and the transmitter is at an unknown location (passive localization). Some practical implementations of passive localization were described in [7,8]. It is important to point out that the methods in [5,6,7,8] relied on spatial coherence for increased accuracy, but are suited to narrowband signals.

In [9,10], it was shown that the accuracy of TDoA estimation could be significantly increased by exploiting the carrier phase of arrival. Since direct localization is implicitly based on TDoAs, this suggests that the localization accuracy could also benefit significantly from exploiting the carrier phases in spatially-coherent scenarios. The 5G of cellular networks is expected to include a wide range of disruptive technologies for large performance improvements. Some of these are millimeter-wave (mmWave) technologies, as described in [11,12,13,14,15,16,17], and massive MIMO systems [18,19]. There are also methods for cooperative localization in cellular systems and approaches to using distributed massive MIMO systems for resolving the ambiguity problem, which is inherent to coherent localization [20].

Coherent localization presents a great promise in 5G systems because it is expected to be carried out with mmWave and in small cells. Further, the experimental results from [11] showed that the non-line-of-sight (NLoS) components were at least 10dB below the line-of-sight (LoS) component in the 60-GHz range. Further, one can expect large bandwidths in 5G systems, which is why it is important to formulate practical methods that extract the information in the carrier phases and that are suited to wideband signals.

In this paper, we introduce methods for direct and coherent localization of transmitters of arbitrary wideband signals by a distributed receiving antenna array (the TDoA between any two of the array’s antennas may be larger than the inverse of the signal bandwidth). The receiving channels are phase and time synchronized, but the alignment of the transmitter’s time axis to the one of the receiving system is not required (unlike for ToA-based methods such as in [6]). In contrast to, for example [5,6], our signal model is both frequency-wideband and spatial-wideband. The effects of spatial-wideband and frequency-wideband signals are discussed in detail in [21,22]. We assume multipath propagation with LoS components. Some of the antennas in the receiving array may be grouped into subarrays of small apertures, but the mathematical model does not use the plane wave approximation across these subarrays (it assumes spherical wavefronts).

The methods in this paper achieve accuracy that is two or three orders of magnitude better than the carrier wavelength. The purpose of having such accuracy in the estimate of a user terminal’s location is to improve drastically the system performance by focusing energy towards the terminal on the down-link and increasing sensitivity on the up-link so that the terminal can transmit at much lower power (the LAC concept).

The main contributions of this paper are as follows. We propose two maximum-likelihood-type (ML) algorithms (for the LoS-only scenario) and one steered covariance matrix-based MUSIC-type (SCM-MUSIC) algorithm for wideband direct position estimation. The methods are proposed for a signal model that is both spatial-wideband and frequency-wideband, according to [21,22]. These two papers discussed the importance of wideband modeling even for collocated massive antenna arrays. As a consequence, wideband modeling is critical in distributed systems. One of the ML algorithms builds on the knowledge of the waveform (sequence), whereas the other two do not. Despite not using this knowledge, these two algorithms are statistically efficient for the LoS-only scenario, which we demonstrate by Monte Carlo simulations. We also show that they perform well even in multipath settings. The algorithms do not exhibit a pronounced threshold effect and achieve accuracy better than the carrier wavelength even for four-sample-long sequences.

The rest of this paper is organized as follows. Section 2 introduces the model of the signals and the system used for localization. In Section 3, we propose two ML and one MUSIC-type estimation algorithm. We discuss the results of Monte Carlo simulations in Section 4 and provide concluding remarks in Section 5. The ML algorithms are derived in the Appendix A.

## 2. System and Signal Model

### 2.1. System Model

**Figure 1:**
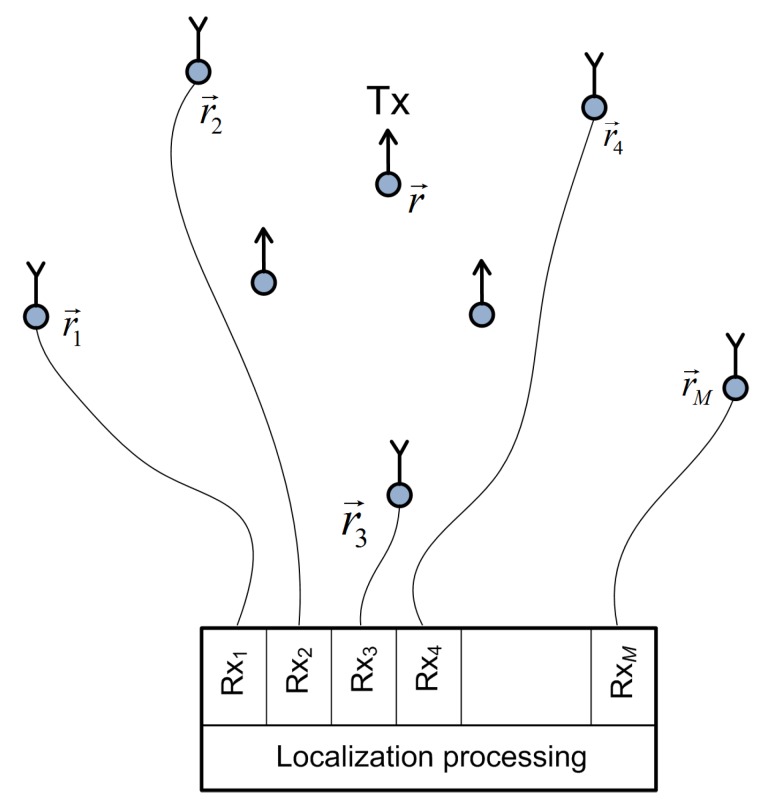
The topology of the studied system.

A distributed receiving antenna array that performs the localization has *M* stationary elements/channels, ${\mathrm{Rx}}_{m}$, $m\in \left\{1,2\ldots M\right\}$, where the antenna ${\mathrm{Rx}}_{m}$ is at a known position ${\vec{r}}_{m}\;=\;\left(x_{m},y_{m},z_{m}\right)$; see Figure 1. An active stationary transmitter, Tx, is at an unknown position $\vec{r}=\left(x,y,z\right)$. All receive and transmit antennas are arbitrarily distributed in the 3D area of interest for which we assume spatial coherence of the LoS components across the entire distributed receiving array. The coherence can be established if the carrier phase difference between any two of the receiving antennas, ${\mathrm{Rx}}_{m}$ and ${\mathrm{Rx}}_{l}$, can be obtained from the difference in the distances between them and the Tx. In mathematical terms, we have:

(1) $$\varphi_{cm}-\varphi_{cl}=2\pi \nu_{c}\left(d_{l}-d_{m}\right)/\tilde{c}\;\;\;\left(\mathrm{mod}\;2\pi \right),$$

where $\varphi_{cm}$ and $\varphi_{cl}$ are the carrier phases of the $m^{\mathrm{th}}$ and $l^{\mathrm{th}}$ receiving antennas, respectively; $\nu_{c}$ is the carrier frequency in Hz; $d_{m}$ is the distance between Tx and ${\mathrm{Rx}}_{m}$ antennas, $d_{m}=∥{\vec{d}}_{m}∥$, ${\vec{d}}_{m}=\vec{r}-{\vec{r}}_{m}$, where ∥·∥ is the Euclidean norm; $\tilde{c}$ is the speed of propagation ($\tilde{c}=3\cdot 10^{8}\;m/s$). The distributed receiving antennas are connected to *M* collocated receiving channels (front-ends) by calibrated cables (coaxial or fiber optic) for two reasons. The first is that fine time, frequency, and phase synchronization between the receiving channels is easier to achieve with collocated than with distributed front-ends. The second reason it that a centralized (specifically, direct or one-step) localization algorithm is a natural choice in this setup. All antennas act as “isotropic”, and their polarizations are aligned. Deviations from this in practice can be taken into account if there are additional attenuations and phase shifts and whose values are determined by the antenna design. The positions of the receiving antennas, ${\vec{r}}_{m}$, are known in the sense that the antennas must be positioned with accuracy greater than the localization accuracy itself (with an error much smaller than the carrier wavelength). In the proposed system model, in practice, some antennas could be used for localization and the others for down-link communication with Tx.

### 2.2. Signal Model

The Tx transmits an RF signal (approximately) band-limited to $\left(\nu_{c}-B/2,\nu_{c}+B/2\right)$, where *B* is the signal bandwidth in Hz, i.e., the complex form of the transmitted sequence/waveform is band-limited to $\left(-B/2,B/2\right)$. The signal received at the ${\mathrm{Rx}}_{m}$ antenna, $m\in \left\{1,2\ldots M\right\}$, which is an attenuated and delayed version of the transmitted signal, after down-conversion and A/D conversion at the Nyquist rate $\nu_{s}=B$, is represented in a complex form ${\bar{u}}_{m}\left(n\right)$ as:

(2) $$\begin{array}{cc} {\bar{u}}_{m}\left(n\right) & =a_{m}s_{m}\left(n\right)+{\bar{u}}_{\mathrm{NLoS}m}\left(n\right)+{\bar{\eta }}_{m}\left(n\right),\\ s_{m}\left(n\right) & =\exp\left(-j\omega_{c}\left(t_{0}+\tau_{m}\right)\right)s\left(n-t_{0}-\tau_{m}\right),\\ {\bar{u}}_{\mathrm{NLoS}m}\left(n\right) & =\sum_{l=1}^{L}a_{m,l}s\left(n-\tau_{m,l}\right), \end{array}$$

where *n* denotes discretized time normalized with $1/\nu_{s}$, i.e., $n\in \left\{0,1\ldots N-1\right\}$; $a_{m}$ is a real-valued attenuation factor for the LoS component; $\omega_{c}=2\pi f_{c}$, $f_{c}=\nu_{c}/B$ are the normalized angular and natural carrier frequencies, respectively; the unknown value $t_{0}$ models the fact that the Tx is not time synchronized with the Rx system (we always use the Rx time axis); the normalized propagation delays from the Tx to the antenna ${\mathrm{Rx}}_{m}$ are $\tau_{m}=d_{m}/c$, where the normalized propagation speed is $c=\tilde{c}/\nu_{s}$; ${\bar{u}}_{\mathrm{NLoS}m}\left(n\right)$ denotes all of the multipath components of the Tx signal except the LoS; ${\bar{\eta }}_{m}\left(n\right)$ is the noise; $a_{m,l}$ is an unknown complex-valued attenuation factor of the $l^{\mathrm{th}}$ component received by the $m^{\mathrm{th}}$ Rx channel, and $\tau_{m,l}$ is an appropriate time delay; the acquired samples are ${\bar{u}}_{m}\left(n\right)$. Note that $t_{0}$, $d_{m}/c$, and $\tau_{m,l}$ are fractional dimensionless values (the propagation time delays do not have to be integer multiples of the sampling interval) and that the sequence/waveform s(·) is defined in the entire (continuous) range R since it has been transformed into a continuous waveform inside the Tx.

A signal model on a large antenna array can be frequency- and spatial-wideband, and the effects of these phenomena are discussed in detail in [21]. The model has to be spatially wideband, if the propagation time across the aperture of the receiving array is greater than the inverse of the signal bandwidth, 1/B. It models envelope shifts between the signals in the receiving channels in addition to carrier phase shifts (Strictly speaking, the signals have to be modeled as spatially wideband, if the approximation $s\left(n-t_{0}-\tau_{m}\right)\approx s\left(n-t_{0}-\tau_{l}\right)$ does not hold for some $m,l\in \left\{1,2\ldots M\right\}$. The signal is spatially narrowband if $s\left(n-t_{0}-\tau_{m}\right)\approx s\left(n-t_{0}-\tau_{l}\right)$, $\left(\forall m,l\in \left\{1,2\ldots M\right\}\right)$.). It is well known that, if the signal model is spatially wideband, then the localization methods have to be formulated in the spectral domain. Our signal model does not limit the signal bandwidth, so, in the context of [21], our model is both frequency-wideband and spatial-wideband (Consider a numerical example in the mmWave band. If a signal with bandwidth B=100MHz at a carrier frequency $\nu_{c}=60\;\mathrm{GHz}$ is transmitted in a conference room equipped with an antenna array with an aperture of, say, 6m, then the propagation time across the array would be two sampling intervals. This shows the importance of wideband modeling.).

We scale the signal ${\bar{u}}_{m}\left(n\right)$ in a preprocessing step by $1/a_{m}$ to obtain $u_{m}\left(n\right)$. We note that the signal-to-noise ratios (SNRs) and noise powers are considered known to the receivers. Thereby, the useful signals in all the receiving channels have the same power. Then, we have:

(3) $$u_{m}\left(n\right)=s_{m}\left(n\right)+u_{\mathrm{NLoS}m}\left(n\right)+\eta_{m}\left(n\right),$$

where $u_{\mathrm{NLoS}m}\left(n\right)={\bar{u}}_{\mathrm{NLoS}m}\left(n\right)/a_{m}$, $\eta_{m}\left(n\right)={\bar{\eta }}_{m}\left(n\right)/a_{m}$, $\eta_{m}\left(n\right)∼\mathrm{CN}\left(0,\sigma_{m}^{2}\right)$ is a circular-complex zero-mean Gaussian random process (the noise) with variance $\sigma_{m}^{2}$, independent across time samples and channels and independent of the useful signal s(t). The variabilities of the propagation attenuations are modeled by $\sigma_{m}^{2}$, but the model considers that no information about the transmitter location is contained in the attenuations. We assumed this because, in practice, location estimation based on phases is more robust than estimation based on attenuations. Additionally, this reduces the number of dimensions of the search grid in the proposed algorithms. However, the algorithms use the SNRs for channel weighting. We define the SNR in channel *m* as ${\mathrm{SNR}}_{m}=1/\left(N\sigma_{m}^{2}\right)\sum_{n=0}^{N-1}{\left|s(n)\right|}^{2}$.

We implement the time shifting of an RF signal by performing operations in the discrete Fourier transform (DFT) domain on its complex envelope, and therefore, strictly speaking, the signal has to be periodic with period *N* (the length of the observation interval). However, this does not have to be the case if the error created by a cyclic time shift, compared to the regular time shift, is negligible, which is important for the random sequence scenario.

If the ${\left(h,q\right)}^{\mathrm{th}}$ entry of a matrix A is $A_{h,q}$, define the ${\left(h,q\right)}^{\mathrm{th}}$ entry of $\exp\left(A\right)$ as $\exp(A_{h,q})$. Furthermore, for any vector v, let $\mathrm{Diag}\left\{v\right\}$ be the diagonal matrix whose elements on the main diagonal are the elements of v. Then, the discrete-time matrix form of the signal model (3) is:

(4) $$\begin{array}{cc} u_{m} & =F^{H}D_{t_{0}+d_{m}/c}Fs+u_{\mathrm{NLoS}m}+\eta_{m}, \end{array}$$

(5) $$\begin{array}{cc} F & =1/\sqrt{N}\exp\left(-j2\pi /N\cdot kn^{⊤}\right), \end{array}$$

(6) $$\begin{array}{cc} D_{\tau } & =\exp\left(-j\omega_{c}\tau \right)\mathrm{Diag}\left\{\exp\left(-j2\pi /N\cdot k\tau \right)\right\}\;\left(\forall \tau \right), \end{array}$$

(7) $$\begin{array}{cc} n & ={\left[0,1,\ldots ,N-1\right]}^{⊤}, \end{array}$$

(8) $$\begin{array}{cc} k & ={\left[-N/2,-N/2+1,\ldots ,N/2-1\right]}^{⊤}, \end{array}$$

where $u_{m}={\left[u_{m}\left(0\right),u_{m}\left(1\right),\ldots ,u_{m}\left(N-1\right)\right]}^{⊤}$, $s={\left[s(0),s(1),\ldots ,s(N-1)\right]}^{⊤}$, and $\eta_{m}\;=\;[\eta_{m}\left(0\right),\eta_{m}\left(1\right),$$\ldots ,\eta_{m}{\left(N-1\right)]}^{⊤}$; F is a modified Fourier transform matrix; $D_{\tau }$ is the delay-by-τ operator, for any τ; ${\left(\cdot \right)}^{⊤}$ and ${\left(\cdot \right)}^{H}$ denote transpose and conjugate transpose, respectively. In the known signal scenario, the conditions that the continuous waveform s(t) is band-limited to $\left(-1/2,1/2\right)$ and that it is periodic with period *N* imply that, given the samples in s, the signal in the entire (continuous) range t∈R is $s\left(t\right)=1/N\sum_{k=-N/2}^{N/2-1}S\left(k\right)\exp\left(j2\pi kt/N\right)$, where $S\left(k\right)=\sum_{n=0}^{N-1}s\left(n\right)\exp\left(-j2\pi kn/N\right)$. In the random signal scenario, s(n), n∈Z, is a circular-complex zero-mean Gaussian random process with variance $\sigma_{s}^{2}$, so that ${\mathrm{SNR}}_{m}=\sigma_{s}^{2}/\sigma_{m}^{2}$.

The goal is to estimate $\vec{r}$ given the observations $u_{m}$, $m\in \left\{1,2\ldots M\right\}$.

## 3. Localization Algorithms

### 3.1. Maximum Likelihood Algorithms

For simplicity, we derive the ML algorithms for ${\bar{u}}_{\mathrm{NLoS}m}\left(n\right)=0$. We later show that they are applicable in multipath propagation conditions as well. If $Q_{m}=F^{H}D_{t_{0}+d_{m}/c}F$ and ${∥\cdot ∥}_{F}$ is the Frobenius norm, then the signal in channel *m* is $u_{m}=Q_{m}s+\eta_{m}$ and the log likelihood function (without the additive constant) multiplied by −1 becomes:

(9) $$f_{\mathrm{LL}}=\sum_{m=1}^{M}\frac{1}{\sigma_{m}^{2}}{∥u_{m}-Q_{m}s∥}_{F}^{2},$$

where s and $\eta_{m}$ are defined in Section 2. According to the ML method, the estimate of the vector of unknowns, $\alpha =\left(t_{0},x,y,z\right)$, is the one that minimizes $f_{\mathrm{LL}}$. We formulate two algorithms.

ML algorithm for a known sequence (ML-KS): If the sequence s is considered known, the estimate of α is the one that maximizes the criterion function:

(10) fKS2=Re∑m=1M1σm2umHQms.

One possible implementation of the ML-KS is given in Algorithm 1. Reliance on the knowledge of the waveform requires that the local carrier is coupled with the A/D (or D/A) converter clock in each receiving and transmitting channel.

ML algorithm for an unknown sequence (ML-US): If s is considered unknown, then the estimate of $\alpha =\left(x,y,z\right)$ (without loss of generality, we can set $t_{0}=0$ in this case) is the one that maximizes the criterion function:

(11) $$f_{\mathrm{US}2}=\sum_{l=1}^{M}\sum_{m=1}^{M}\frac{1}{\sigma_{l}^{2}\sigma_{m}^{2}}u_{l}^{H}F^{H}D_{d_{l}/c-d_{m}/c}Fu_{m}.$$

The derivation details are provided in the Appendix A and an implementation in Algorithm 2.

The criterion function $f_{\mathrm{US}2}$ can also be written in the following matrix form. The weighted signals in the DFT domain are given by $U_{m}^{\prime }=w_{m}Fu_{m}$, where the weighting coefficients are $w_{m}=1/\sigma_{m}^{2}$, $m\in \left\{1,2,\ldots ,M\right\}$. Let the signals compensated for the propagation delays for a hypothetical Tx location in the search grid, $\vec{r}$, be $U_{m,\mathrm{comp}}=D_{\tau_{\mathrm{CM}}-d_{m}/c}U_{m}^{\prime }$, where $\tau_{\mathrm{CM}}$ is arbitrarily chosen and constant across the channels. To reduce numerical errors, we define $\tau_{\mathrm{CM}}$ as the mean (center of mass) of $d_{m}/c$, $m\in \left\{1,2,\ldots ,M\right\}$. If $U_{\mathrm{comp}}=\left[U_{1,\mathrm{comp}},U_{2,\mathrm{comp}},\ldots ,U_{M,\mathrm{comp}}\right]$, the steered covariance matrix can be written as $R\left(\vec{r}\right)=U_{\mathrm{comp}}^{H}U_{\mathrm{comp}}\in C^{M\times M}$. Finally, we have $f_{\mathrm{US}2}=v^{H}R\left(\vec{r}\right)v$, where v is a column vector composed of elements equal to one.

Note that the ML-US algorithm cannot estimate $t_{0}$, since this is only possible if it knows the sequence. This, however, reduces the number of dimensions in the search grid by one. The product $Fu_{m}$ is the DFT spectrum of the signal in channel *m*, and $Fu_{m}/\sigma_{m}^{2}$ can be calculated only once per channel during the preprocessing to optimize the algorithm numerically. If an algorithm does not rely on the knowledge of the waveform, the requirements for the transmitter hardware are relaxed, so that any off-the-shelf transmitter can be localized. Furthermore, the applications of this type of localization also include non-cooperative cases.

**Algorithm 1** An implementation of the ML-KS algorithm
  1.**input**${\vec{r}}_{Ti}$ ($N_{p}$ position vectors for hypothetical transmitter locations in the search grid), $t_{0}$ (a vector with $N_{t_{0}}$ values for $t_{0}$ to be searched for), *c*, *M*, *N*, $f_{c}$, s, ${\vec{r}}_{m}$, $\sigma_{m}$, $u_{m}$, for $m\in \left\{1,2,\ldots ,M\right\}$  2.Calculate the spectrum of the known sequence s, S=Fs  3.**for** each gridpoint along the $t_{0}$ dimension of the grid, j=1 to $N_{t_{0}}$ **do**  4. Calculate $D_{t_{0}\left(j\right)}$ according to (6)  5.
**end for**
  6.**for** each Rx antenna, m=1 to *M* **do**  7. $U_{m}=\frac{1}{\sigma_{m}^{2}}Fu_{m}$
  8.
**end for**
  9.**for** each gridpoint along the spatial dimensions of the grid, i=1 to $N_{p}$ **do**10. **for** each Rx antenna, m=1 to *M* **do**11.  $d_{m}=∥{\vec{r}}_{m}-{\vec{r}}_{Ti}∥$
12.  Calculate $D_{d_{m}/c}$ according to (6)13. **end for**
14. **for** each gridpoint along the $t_{0}$ dimension of the grid, j=1 to $N_{t_{0}}$ **do**15.  Calculate the criterion function fij=∑m=1MRe(UmHDdm/cDt0(j)S)16. **end for**
17.
**end for**
18.
$\left(i_{0},j_{0}\right)=\underset{i,j}{\arg\;\max\;}\;f_{ij}$
19.
**return** $\hat{\vec{r}}={\vec{r}}_{Ti_{0}},{\hat{t}}_{0}=t_{0}\left(j_{0}\right)$



**Algorithm 2** An implementation of the ML-US algorithm
  1.**input**${\vec{r}}_{Ti}$ ($N_{p}$ position vectors for hypothetical transmitter locations in the search grid), *c*, *M*, *N*, $f_{c}$, ${\vec{r}}_{m}$, $\sigma_{m}$, $u_{m}$, for $m\in \left\{1,2,\ldots ,M\right\}$  2.**for** each Rx antenna, m=1 to *M* **do**  3. $U_{m}=\frac{1}{\sigma_{m}^{2}}Fu_{m}$
  4.
**end for**
  5.**for** each gridpoint along the spatial dimensions of the grid, i=1 to $N_{p}$ **do**  6. **for** each Rx antenna, m=1 to *M* **do**  7.  $d_{m}=∥{\vec{r}}_{m}-{\vec{r}}_{Ti}∥$
  8.  Calculate $D_{d_{m}/c}$ according to (6)  9. **end for**
10. Calculate the criterion function fi=∑l=2M∑m=1l−1Re(UlHDdl/cDdm/cHUm)11.
**end for**
12.
$i_{0}=\underset{i}{\arg\;\max\;}\;f_{i}$
13.
**return** $\hat{\vec{r}}={\vec{r}}_{Ti_{0}}$



Note that the identities $D_{d_{m}/c+t_{0}\left(j\right)}=D_{d_{m}/c}D_{t_{0}\left(j\right)}$ and $D_{d_{l}/c-d_{m}/c}=D_{d_{l}/c}D_{d_{m}/c}^{H}$ were used to optimize the algorithms numerically. Furthermore, note that S=Fs is equivalent to the command S = fftshift(fft(s))/sqrt(N) in MATLAB.

### 3.2. Steered Covariance Matrix-Based MUSIC-Type Algorithm

A method for AoA estimation of wideband acoustic signals based on a steered covariance matrix approach was proposed in [23]. The method is more efficient for short observation intervals than the spectral focusing AoA approach proposed in [24]. A method for direct position estimation of baseband UWB signals was proposed in [25]. It is based on the steered covariance matrix approach as well.

In this paper, we generalize the steered covariance matrix approach for direct position estimation of wideband bandpass radio signals by introducing the SCM-MUSIC algorithm. This algorithm uses preprocessed signals defined by $U_{m}^{\prime }=w_{m}Fu_{m}$, where $w_{m}=1/\sigma_{m}$. This means that, after this step, the noise powers are equal across the channels. Starting from $U_{m}^{\prime }$, the steered covariance matrix, $R\left(\vec{r}\right)$, is obtained in the same way as by the ML-US algorithm. The noise subspace matrix $E_{n}\left(\vec{r}\right)\in C^{M\times (M-1)}$ is formed from $R\left(\vec{r}\right)$ in the same way as in [23,26]. Namely, it is a block matrix, $E_{n}\left(\vec{r}\right)=\left[v_{e2},v_{e3},\ldots ,v_{eM}\right]$, where $v_{em}$ is the norm-one eigenvector of $R\left(\vec{r}\right)$ corresponding to its eigenvalue $\lambda_{m}$, where $\lambda_{1}\geq \lambda_{2}\geq \ldots \geq \lambda_{M}$. The matrix has M−1 columns when there is a single active transmitter. The estimated location is the one that maximizes the criterion function:

(12) $$f_{\mathrm{SCM}-\mathrm{MUSIC}}=\frac{v^{H}v}{v^{H}E_{n}\left(\vec{r}\right)E_{n}^{H}\left(\vec{r}\right)v},$$

where $v={\left[1/\sigma_{1},1/\sigma_{2},\ldots ,1/\sigma_{M}\right]}^{⊤}$. Algorithm 3 provides an implementation of the SCM-MUSIC.

Even though the criterion function (12) has a similar form as the original MUSIC in [26], the differences of the SCM-MUSIC algorithm relative to the ones in the literature are as follows. In our approach, the unknown parameters (the location of the Tx) are contained in the covariance matrix (this is the steered covariance matrix approach), whereas in [5,26], they were contained in the steering vector, which limits the application to narrowband signals. We model bandpass signals, unlike [23,25], where the signals are in the baseband ([23] is for acoustic signals), and our antenna array is distributed, unlike [23,26]. Instead of changing the attenuations over the search grid, we used channel weights based on the received SNRs, unlike [5,25].

**Algorithm 3** An implementation of the SCM-MUSIC algorithm
  1.**input**${\vec{r}}_{Ti}$ ($N_{p}$ position vectors for hypothetical transmitter locations in the search grid), *c*, *M*, *N*, $f_{c}$, ${\vec{r}}_{m}$, $\sigma_{m}$, $u_{m}$, for $m\in \left\{1,2,\ldots ,M\right\}$  2.Calculate $v={\left[1/\sigma_{1},1/\sigma_{2},\ldots ,1/\sigma_{M}\right]}^{⊤}$  3.**for** each Rx antenna, m=1 to *M* **do**  4. $w_{m}=1/\sigma_{m}$
  5. $U_{m}^{\prime }=w_{m}Fu_{m}$
  6.
**end for**
  7.**for** each gridpoint along the spatial dimensions of the grid, i=1 to $N_{p}$ **do**  8. **for** each Rx antenna, m=1 to *M* **do**  9.  $d_{m}=∥{\vec{r}}_{m}-{\vec{r}}_{Ti}∥$
10. **end for**
11. Calculate the steered-covariance matrix R12. Perform eigendecomposition of R to obtain $v_{em}$, $m\in \left\{1,2,\ldots ,M\right\}$13. Form the noise subspace matrix $E_{n}=\left[v_{e2},v_{e3},\ldots ,v_{eM}\right]$14. Calculate the criterion function $f_{i}=1/\left(v^{H}E_{n}E_{n}^{H}v\right)$15.
**end for**
16.
$i_{0}=\underset{i}{\arg\;\max\;}\;f_{i}$
17.
**return** $\hat{\vec{r}}={\vec{r}}_{Ti_{0}}$



Note that $v_{em}$ is the norm-one eigenvector of R corresponding to its eigenvalue $\lambda_{m}$, where $\lambda_{1}\geq \lambda_{2}\geq \ldots \geq \lambda_{M}$.

### 3.3. Noncoherent Algorithms

The algorithms proposed in this section are computationally intensive when applied to the entire distributed array, and so, we can adopt a suboptimal method to narrow the search grid first. We applied the idea used in [3] to define the maximum covariance matrix eigenvalue (MCME) algorithm. Its criterion function is equal to the largest eigenvalue of the covariance matrix $R\left(\vec{r}\right)$ defined for the previous algorithms, as opposed to the criterion function in (11), which is equal to the sum of the elements of $R\left(\vec{r}\right)$. The MCME algorithm does not use the spatial coherence between the signals received by the distributed antenna array.

### 3.4. Numerical Complexity of the Algorithms

We now address the number of operations, NO, of the proposed algorithms. We write:

(13) $$\mathrm{NO}={\mathrm{NO}}_{\mathrm{PP}}+\frac{A}{Q}\times {\mathrm{NO}}_{\mathrm{GP}},$$

where ${\mathrm{NO}}_{\mathrm{PP}}$ is the number of operations in the preprocessing step, A/Q is the number of points in the search grid, and ${\mathrm{NO}}_{\mathrm{GP}}$ is the number of operations for a single grid point. The size of the area that the search grid spans is *A*, which is either a surface for 2D or a volume for 3D localization. The resolution cell of the grid is Q=ΔxΔy or Q=ΔxΔyΔz (2D or 3D). A coherent algorithm requires a very fine grid, so that Δx, Δy, and Δz are in the order of $\lambda_{c}/1000$, whereas a noncoherent algorithm can work with a much coarser grid, where Δx, Δy, and Δz are in the order of $\tilde{c}/B/1000$. Thanks to the FFT (fast Fourier transform) algorithm, we have that ${\mathrm{NO}}_{\mathrm{PP}}=M\times \left(\left(1+N/2\log_{2}N\right)m_{C}+N\log_{2}Na_{C}\right)$, where $m_{C}$ is a single complex multiplication and $a_{C}$ is a single complex addition. The number of operations in the preprocessing step for coherent methods is expected to be negligible compared to the search over the grid, because of a large number of grid points. Further, we have that each grid point requires:

(14) $${\mathrm{NO}}_{\mathrm{GP}}=M\times {\mathrm{NO}}_{\mathrm{MD}}+{\mathrm{NO}}_{\mathrm{AS}}$$

operations, where ${\mathrm{NO}}_{\mathrm{MD}}$ is the number of operations for the calculation of the main diagonal of the matrix $D_{d_{m}/c}$, and ${\mathrm{NO}}_{\mathrm{AS}}$ is an algorithm-specific value, which for the ML-KS algorithm is:

(15) $${\mathrm{NO}}_{\mathrm{AS}}=N_{t_{0}}\left(M\left(3Nm_{C}+Na_{C}\right)-a_{C}\right),$$

where $N_{t_{0}}$ is the number of points along the $t_{0}$ dimension of the search grid, for the ML-US algorithm is:

(16) $${\mathrm{NO}}_{\mathrm{AS}}=MNm_{C}+\frac{M(M-1)}{2}N\left(m_{C}+a_{C}\right)-a_{C},$$

and for the SCM-MUSIC algorithm, based on the numerical complexity in [27], is:

(17) $${\mathrm{NO}}_{\mathrm{AS}}\approx MNm_{C}+\frac{M(M-1)}{2}\left(Nm_{C}+\left(N+1\right)a_{C}\right)+\frac{13}{3}M^{3}\left(m_{C}+a_{C}\right)+M^{2}m_{C}.$$

Note that the two extreme cases for the matrix $D_{d_{m}/c}$ are to either calculate it each time or to read it from a memory, which has been filled in advance with values calculated offline for the entire grid. There are also possibilities in between these two extreme cases. Furthermore, note that, when the signals are narrowband, in the limit, multiplying a signal vector by $D_{\tau }$ reduces to multiplying the vector by a simple scalar $\exp\left(-j\omega_{c}\tau \right)$. In the general wideband case and when the matrix $D_{d_{m}/c}$ is calculated each time, we have that ${\mathrm{NO}}_{\mathrm{MD}}=Nm_{C}+Ne_{C}$, where $e_{C}$ is a single (scalar) complex exponential function.

All in all, the approximate numerical complexity of the ML-KS, ML-US, and SCM-MUSIC algorithms, respectively, is given by:

$$\begin{array}{cc} {\mathrm{NO}}^{\mathrm{ML}-\mathrm{KS}}\approx & M\log_{2}N\left(N/2m_{C}+Na_{C}\right) \end{array}$$

(18) $$\begin{array}{cc} & +\frac{A}{Q}\left(MNm_{C}+MNe_{C}+N_{t_{0}}\left(M\left(Na_{C}+3Nm_{C}\right)-a_{C}\right)\right),\\ {\mathrm{NO}}^{\mathrm{ML}-\mathrm{US}}\approx & M\log_{2}N\left(N/2m_{C}+Na_{C}\right) \end{array}$$

(19) $$\begin{array}{cc} & +\frac{A}{Q}\left(2MNm_{C}+MNe_{C}+\frac{M(M-1)}{2}N\left(m_{C}+a_{C}\right)\right),\\ {\mathrm{NO}}^{\mathrm{SCM}-\mathrm{MUSIC}}\approx & M\log_{2}N\left(N/2m_{C}+Na_{C}\right)\\ & +\frac{A}{Q}\left(2MNm_{C}+MNe_{C}+\frac{M(M-1)}{2}\left(Nm_{C}+\left(N+1\right)a_{C}\right)\right. \end{array}$$

(20) $$\begin{array}{cc} & \left.+\frac{13}{3}M^{3}\left(m_{C}+a_{C}\right)+M^{2}m_{C}\right). \end{array}$$

Table 1 shows approximate values of the numerical complexity of the proposed methods for a specific example with parameters M=5, N=64, $\nu_{c}=60\;\mathrm{GHz}$, i.e., $\lambda_{c}=5\;\mathrm{mm}$; the resolution cell along the $t_{0}$ dimension of the grid (for ML-KS) is $1/\left(1000\nu_{c}\right)$, and the span of these $t_{0}$ values is the propagation time across an array aperture of 6m (an indoor scenario). The numerical complexity of the preprocessing step, which is executed before the grid search (thus, it is independent of the number of points on the grid), is 960 multiplications and 1920 additions. The table shows the numerical complexity of the search for a single grid point, and as expected, the preprocessing step has negligible numerical complexity because the number of points in the search grid is typically large. The table also shows that ML-KS has a much higher numerical complexity than ML-US because of the search for $t_{0}$. Further, note that SCM-MUSIC has a higher complexity than ML-US, but their complexities are still comparable.

We can reduce the numerical complexity of ML-KS in the following way. Since $t_{0}$ is expected to be much larger than the propagation times, $d_{m}/c$, we can use the procedure explained in [20] (Equations (20)–(23) and the explanations provided with them) to find the total delay ($t_{0}+d_{m}/c$) of the known sequence relative to the beginning of the observation interval by performing a 1D search for each Rx channel. In this way, we can reduce the span of possible $t_{0}$ values from $\left[0,N\right)$ (or $\left[-N/2,N/2\right)$) to an interval as short as the propagation time across the aperture of the receiving array.

We point out that there are other more sophisticated methods for numerical optimization of the cost function and, thus, for localization. One such iterative method, which combines the gradient-descent and particle filters, efficiently searches for the maximum of an ML criterion function, but it does not guarantee finding the global maximum [28]. Investigating optimization methods for maximizing the likelihood functions from Section 3.1 is outside the scope of the paper.

Since a coherent algorithm requires a very fine grid, evaluating its criterion function over the entire area *A* of interest may be prohibitively complex. Using the mentioned idea to narrow down the search grid first by a noncoherent algorithm, we get a reduced number of operations, ${\mathrm{NO}}_{\mathrm{RDC}}$, as:

(21) $${\mathrm{NO}}_{\mathrm{RDC}}={\mathrm{NO}}_{\mathrm{PP}}+\frac{A}{Q_{\mathrm{NCOH}}}\times {\mathrm{NO}}_{\mathrm{GP},\mathrm{NCOH}}+\frac{A_{\mathrm{RDC}}}{Q_{\mathrm{COH}}}\times {\mathrm{NO}}_{\mathrm{GP},\mathrm{COH}},$$

where $Q_{\mathrm{NCOH}}$ and $Q_{\mathrm{COH}}$ are the resolution cells for the noncoherent and coherent algorithms, respectively, ${\mathrm{NO}}_{\mathrm{GP},\mathrm{NCOH}}$ and ${\mathrm{NO}}_{\mathrm{GP},\mathrm{COH}}$ are the appropriate numbers of operations for each grid point, and $A_{\mathrm{RDC}}$ is, for example, a 0.95-confidence interval around the noncoherent location estimate. A coarse grid spans the entire area *A*, whereas a fine grid spans a much smaller grid $A_{\mathrm{RDC}}$.

## 4. Simulations

This section presents numerical results of Monte Carlo simulations performed using the algorithms explained in Section 3 and three distributed receiver antenna array geometries. Throughout this section, we assume that the power of the LoS components decreases with the squared distance from the transmitter. The carrier frequency is $\nu_{c}=60\;\mathrm{GHz}$.

### 4.1. Experimental Setup and Criterion Functions

**Figure 2:**
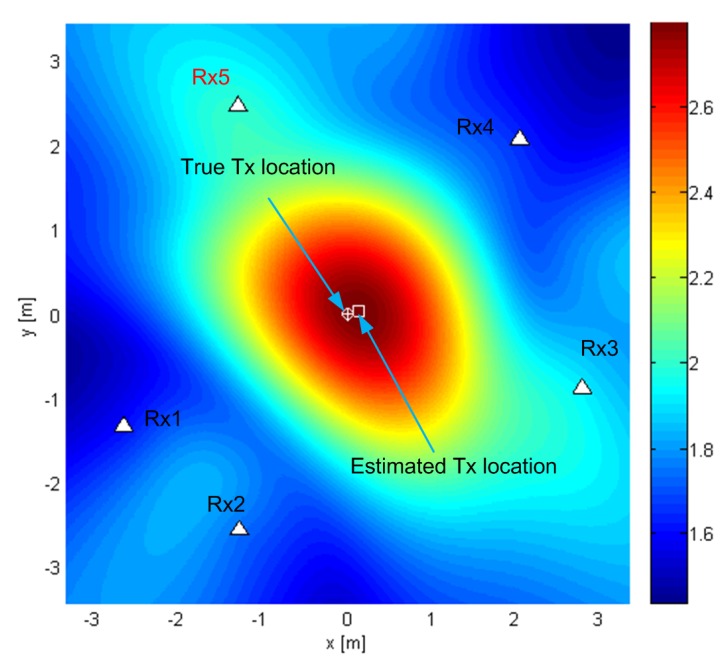
The criterion function for $G_{5}$ and $T_{1}$ for the MCME algorithm.

**Figure 3:**
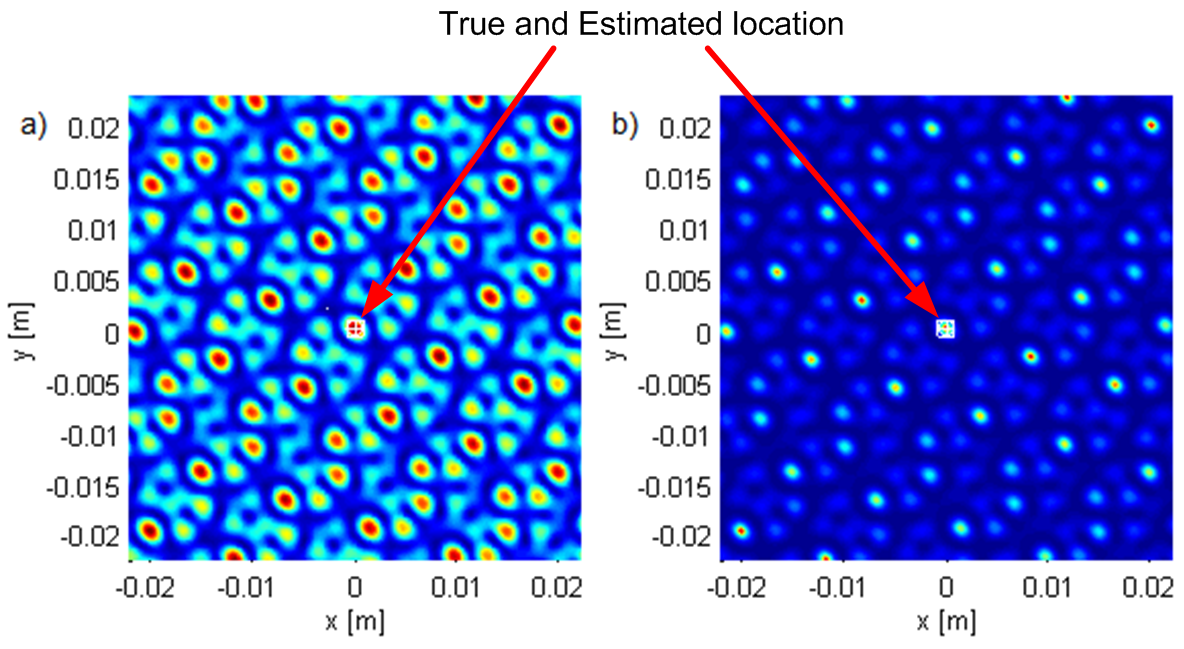
The criterion functions for $G_{5}$ and $T_{1}$ for (**a**) ML-US (magnitude in linear scale) and (**b**) SCM-MUSIC (magnitude in logarithmic scale).

**Figure 4:**
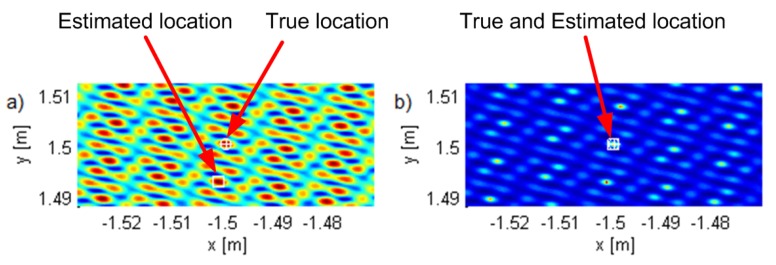
The criterion functions for $G_{5}$ and $T_{2}$ for (**a**) ML-US (magnitude in linear scale) and (**b**) SCM-MUSIC (magnitude in logarithmic scale).

Geometry $G_{5}$ consists of five antennas in the horizontal plane z=0 (see Figure 2) with *x* and *y* coordinates of the antennas in meters (−2.65,−1.32), (−1.28,−2.55), (2.77,−0.87), (2.04,2.07), and (−1.30,2.46), represented by white triangles in the figures. The geometry was chosen by hand in order to be irregular. Figure 2, Figure 3 and Figure 4 show the criterion functions for different Tx locations, random Gaussian signals, and parameters B=100MHz, and N=64. The true Tx location is marked by a circle with a cross and the estimated location by a square. The same colormap was used for all of the displayed criterion functions.

The criterion function of the MCME method is shown in Figure 2 for $G_{5}$, $T_{1}=\left(0,0,0\right)$, ${\mathrm{SNR}}_{0}=10\;\mathrm{dB}$ (which means the SNRs in the channels were approximately 1dB). This particular criterion function is not influenced by carrier phases, has no side lobes, is immune to phase synchronization errors, and varies slowly across space, and so, a coarse grid can be used. This method offers relatively low accuracy (comparable to $\tilde{c}/\nu_{s}$). It is useful for selecting a smaller and finer grid for the other criterion functions in this paper to reduce their computational complexity.

Figure 3 and Figure 4 show the criterion functions for ML-US and SCM-MUSIC, for $G_{5}$, ${\mathrm{SNR}}_{0}\;=\;10\;\mathrm{dB}$, over a small area around the Tx at two characteristic locations, $T_{1}$, randomly chosen near the “center”, and $T_{2}=\left(-1.5,1.5,0\right)$, randomly chosen near the edge of the array, respectively. The main lobes (the lobes where the true locations are) are marked in these figures. The other lobes are side lobes, and they represent the ambiguity problem, which is inherent to coherent methods. The distance between adjacent lobes is in the order of the carrier wavelength, $\lambda_{c}$. These two algorithms have lobes at the same locations; however, the SCM-MUSIC criterion function contains narrower lobes and lower side lobes. Note that the array is relatively inexpensive in terms of hardware and signal processing, but it suffers from the ambiguity problem, which can be seen in Figure 4a; the estimated location (denoted by the white square) is at one of the side lobes in this particular Monte Carlo run. On the other hand, the estimates in Figure 3a,b and Figure 4b were within the main lobe. If a side lobe has approximately the same magnitude as the main lobe, we call it a grating lobe. There is a non-negligible probability that an estimate would be within a grating lobe, as a result of the ambiguity problem. Figure 3 and Figure 4 show the criterion functions over small areas (the criterion functions are zoomed-in) so that the lobe structure can be seen. Note that the lobe structure is determined by the geometry of the receiving array.

If the main lobe is at a point A, a grating lobe appears at any other point B that has the same relative (between the Rx antennas) propagation distances modulo $\lambda_{c}$ as a point A. More formally, if $d_{Am}$ is the distance between A and the ${\mathrm{Rx}}_{m}$ antenna and similarly for $d_{Bm}$, then a grating lobe appears at B if $\left(d_{Am}-d_{A1}-d_{Bm}+d_{B1}\right)/\lambda_{c}\in Z\;\left(\forall m\right)$. As a result, the side lobe patterns are different for $T_{1}$ and $T_{2}$, as can be seen in Figure 3 and Figure 4.

**Figure 5:**
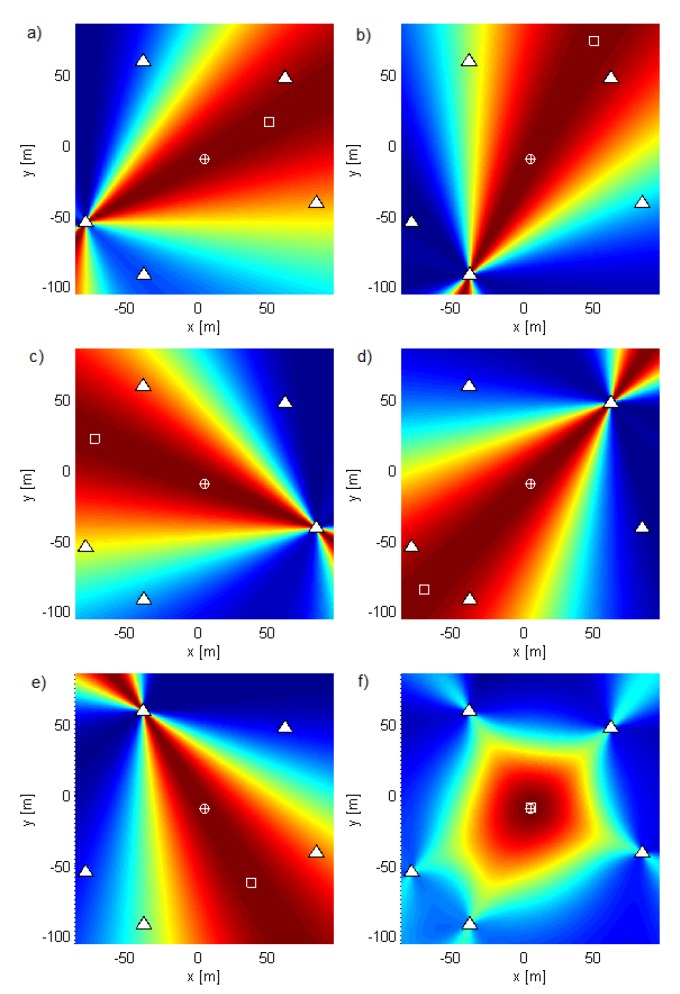
ML-US criterion functions for $f_{c}=4500$ ($\nu_{c}=900\;\mathrm{MHz}$) and $\nu_{s}=200\;\mathrm{kHz}$ applied to (**a**–**e**) individual two-element subarrays in $G_{10}$ and (**f**) the sum of these functions.

**Figure 6:**
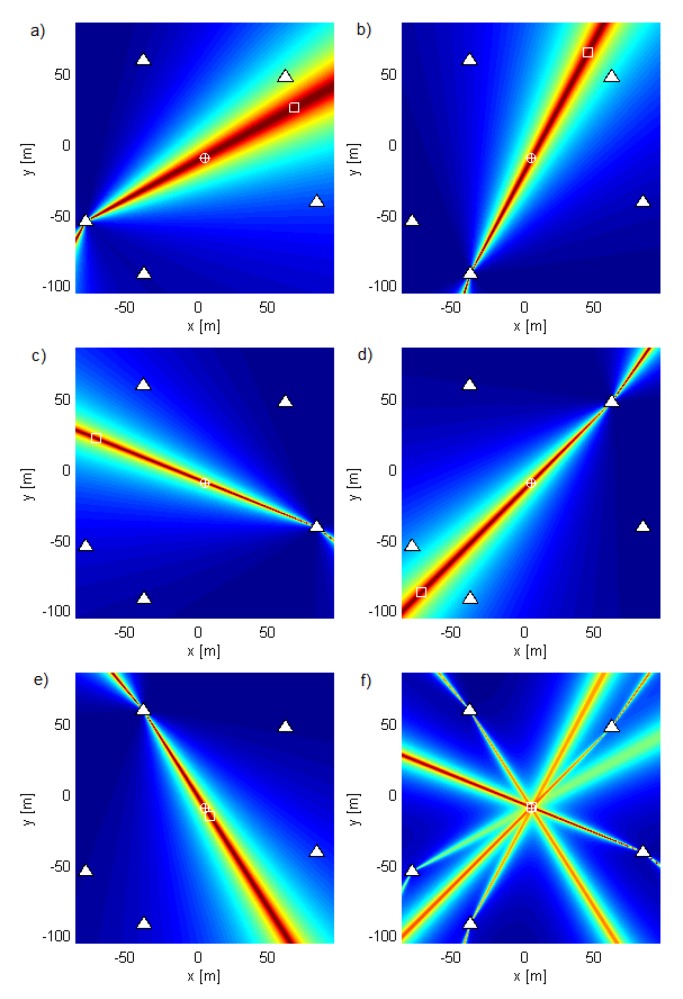
SCM-MUSIC criterion functions for $f_{c}=4500$ ($\nu_{c}=900\;\mathrm{MHz}$) and $\nu_{s}=200\;\mathrm{kHz}$ applied to (**a**–**e**) individual two-element subarrays in $G_{10}$ and (**f**) the sum of these functions.

The geometry $G_{10}$ consists of five antenna subarrays of two antennas separated by $\lambda_{c}/2$ at $\nu_{c}=900\;\mathrm{MHz}$, with *x* and *y* coordinates in meters (−79.49,−54.68), (−79.37,−54.8), (−38.48,−91.52), (−38.33,−91.58), (83.18,−41.23), (83.26,−41.09)(61.06,47.08), (61.19,46.97), (−38.94,58.77), and (−38.81,58.87). The aperture of this geometry is large and so is suitable for outdoor environments. We applied coherent methods to each subarray and then summed the criterion functions of each subarray. As we did not require the subarrays to be phase synchronized with each other, we called the resulting method a semicoherent method. Figure 5 shows the subarray criterion functions in (a–e) and their sum for the ML-US algorithm in (f). Similar results, but for the SCM-MUSIC algorithm, are shown in Figure 6. The criterion functions of these methods have no side lobes; they are not influenced by carrier phases or phase synchronization errors between different subarrays; and they vary relatively slowly across space. Consequently, we can use coarse grids in exploring them. The accuracy of the semicoherent variant is comparable to that of the DPD, [3] and is lower than the accuracy of coherent localization. Despite having lower accuracy, semicoherent localization methods can be used to solve the ambiguity problem and to optimize the search grid (to reduce numerical complexity) of the coherent methods (as previously explained for noncoherent methods). Note that the criterion functions of subarrays with collocated antennas show that the localization methods act as AoA estimation methods.

The metric we used to evaluate the accuracy of the algorithms was the mean squared miss distance (the distance between the estimated location and the true location of the transmitter), i.e., MSE. We also used the RMSE.

**Figure 7:**
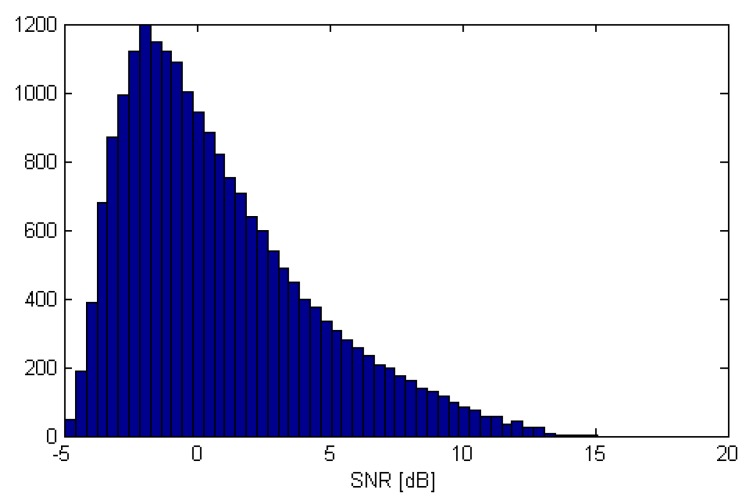
Distribution of SNRs at the antennas for simulated Tx locations for ${\mathrm{SNR}}_{0}=10\;\mathrm{dB}$.

Figure 7 shows the summary distribution of the SNRs at antennas of the array $G_{5}$ for all simulated transmitter locations (Tx grid points) for ${\mathrm{SNR}}_{0}=10\;\mathrm{dB}$. The figure shows that the actual SNRs in most of the channels were between −5 and 10dB.

### 4.2. Results

**Figure 8:**
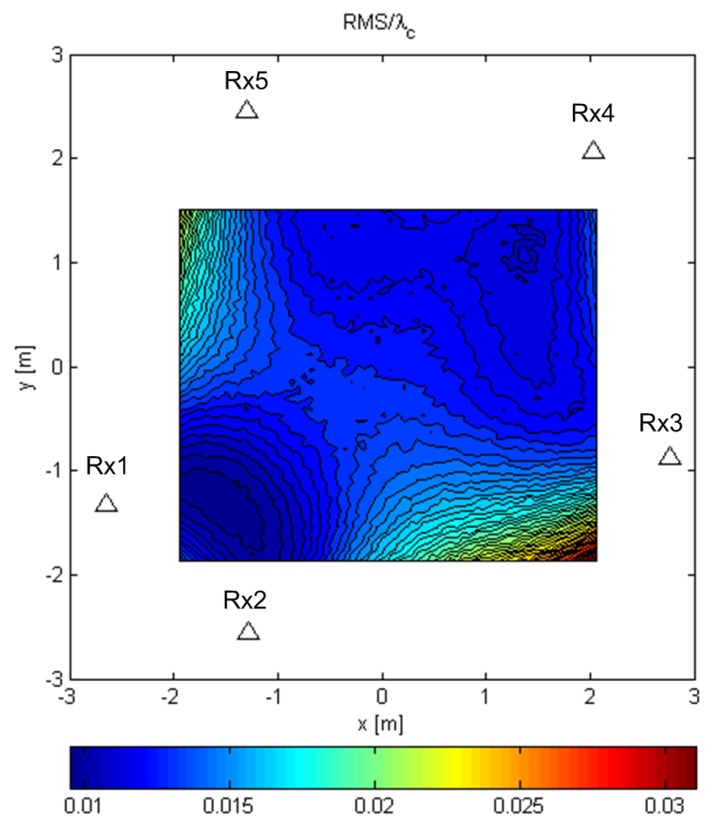
RMSE/$\lambda_{c}$ of the ML-US for random Gaussian signals.

**Figure 9:**
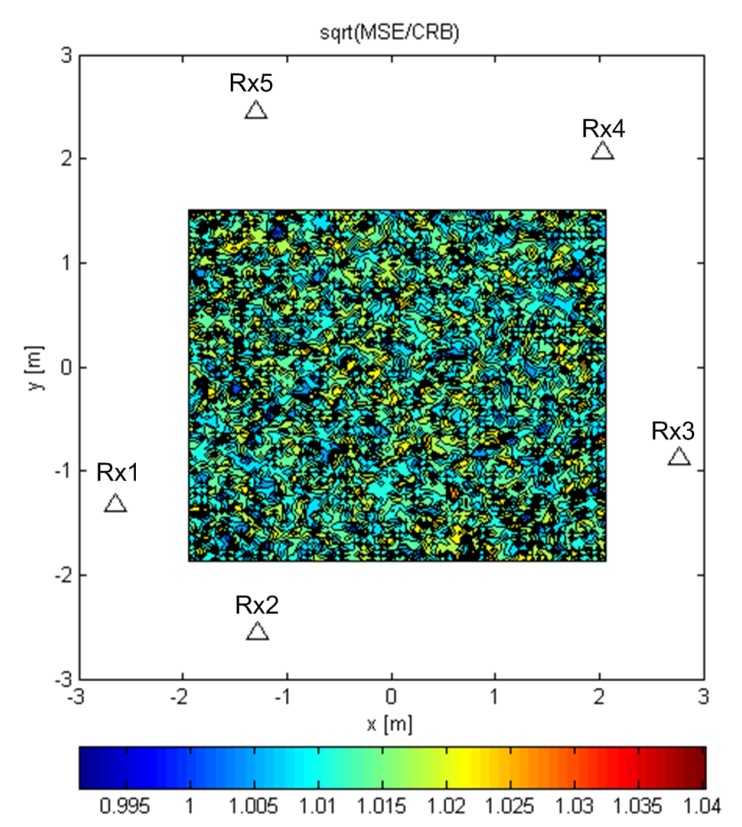
ML-US MSE/CRB for random Gaussian signals.

**Figure 10:**
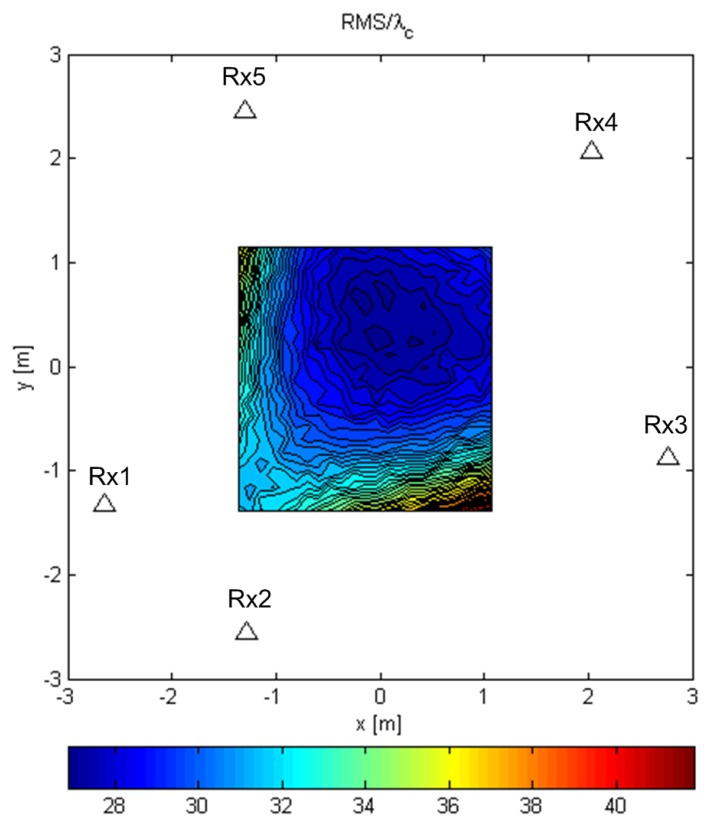
MCME RMSE/$\lambda_{c}$ for random Gaussian signals.

The contour plots in Figure 8, Figure 9 and Figure 10 were generated over Tx grids of uniformly-spaced points in the plane z=0. For every Tx grid point, we performed 8192 Monte Carlo runs. The parameters were B=100MHz, ${\mathrm{SNR}}_{0}=10\;\mathrm{dB}$, and N=64. The simulations were carried out using realizations of a random Gaussian process or a known deterministic sequence, the first of the modulatable orthogonal sequences proposed in [29] for a given *N*. However, the results for the deterministic sequence scenario are not shown for brevity, as they were very similar to those for the random Gaussian signal scenario.

Figure 8 and Figure 9 show the results for the ML-US algorithm over a Tx grid of 64×64 points that covers most of the area inside the array. Figure 8 shows the RMSE (RMS error or the RMS of the distance between the estimated and the true location) values normalized by the carrier wavelength, $\lambda_{c}$, for the random Gaussian signal scenario. For this relatively low SNR and fairly short sequence length, an accuracy of two orders of magnitude better than $\lambda_{c}$ can be achieved. As expected, the accuracy was higher in the inner part of the array aperture and lower near the edge of the aperture. Nevertheless, the accuracy was much better than the carrier wavelength in the entire area in Figure 8. Figure 9 shows the statistical efficiency of the algorithm (the MSE-to-CRB ratio; CRB, Cramér–Rao bound) for the random Gaussian signal scenario. We observed that the efficiency did not depend on the Tx location on the grid and that it was close to one. The figure shows a slight variation over space in the interval $\left[0.995,1.04\right]$, as can be seen from the color bar, but this was due to the randomness in the simulations. The results for the SCM-MUSIC algorithm were very similar to those of the ML-US algorithm.

Figure 10 shows the RMSE values relative to $\lambda_{c}$, for the random Gaussian signal scenario for the MCME algorithm, over a 23×23 grid that covers a smaller area compared to the one used for the ML and SCM-MUSIC algorithms. This is because MCME starts to behave as an AoA estimation algorithm for some Tx locations near the edge of the array. In that case, the miss distance is not an appropriate metric, and so, we chose not to evaluate it in those areas. The RMSE values inside the array were much greater than for the previous algorithms (they exceeded $27\lambda_{c}$ inside the array), because this algorithm did not use the information contained in the carrier phase. However, we can use this criterion function to perform computations over a coarse grid before exploring another criterion function over a finer grid, as explained at the end of the numerical complexity Section 3.4. This accuracy was around $0.04\tilde{c}/\nu_{s}$, which was similar to the accuracy of the conventional methods (those that did not use the carrier phase differences between the received signals).

**Figure 11:**
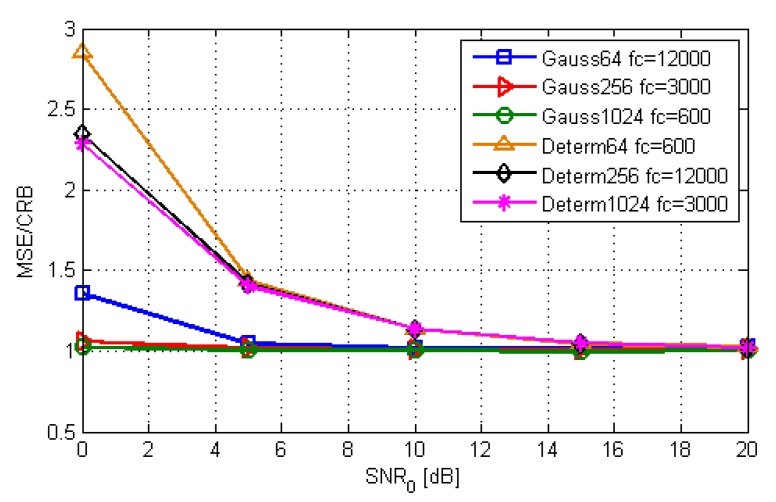
Statistical efficiency of the ML-US as a function of ${\mathrm{SNR}}_{0}$.

The statistical efficiency of the ML-US, averaged over a grid of 32×32 uniformly-distributed points covering an area similar to the one in Figure 8, versus ${\mathrm{SNR}}_{0}$, is shown in Figure 11. The curves are shown for different combinations of sequence length N∈{64,256,1024} and normalized frequency $f_{c}\in \left\{600,3000,12000\right\}$, i.e., B∈{100,20,5}MHz, for both deterministic and random Gaussian signals. For the ML-US algorithm, the statistical efficiency for the random Gaussian scenario was close to one in the observed SNR range, whereas for the deterministic scenario, it started to diverge at lower SNRs in the given range. The results for the SCM-MUSIC algorithm are not shown because its statistical efficiency was similar to that of the ML-US in the observed SNR range, and in the worst case, it was by about 15% higher.

**Figure 12:**
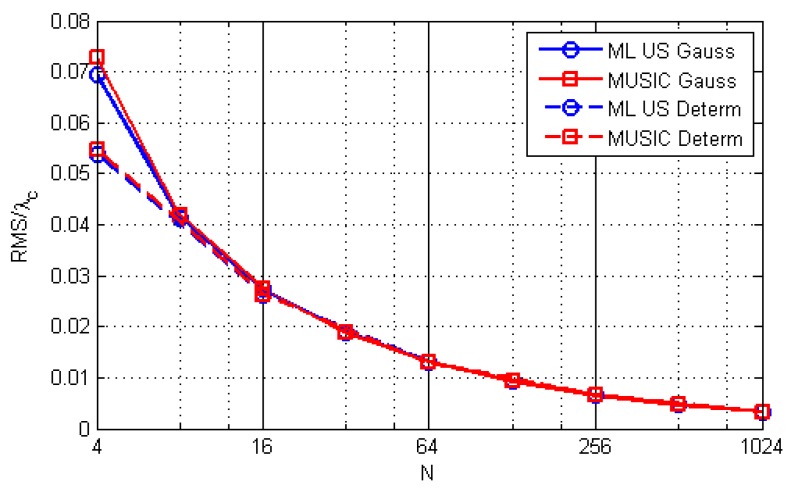
The RMSE/$\lambda_{c}$ vs. *N* for different methods and scenarios.

Figure 12 shows the performance of the algorithms for $f_{c}=600$, ${\mathrm{SNR}}_{0}=10\;\mathrm{dB}$, and different sequence lengths, averaged over the same area as for Figure 11. The curves do not feature a pronounced threshold. This means that even short sequences can be used for localization, which enables spectrum sensing to have a quick response.

We finally point out that in the respective experiments, we worked with other Rx configurations that surrounded the Tx by placing the Rx antennas at different locations and obtained results that were no different than the ones already presented.

### 4.3. Experiments with Subarrays of Antennas

**Figure 13:**
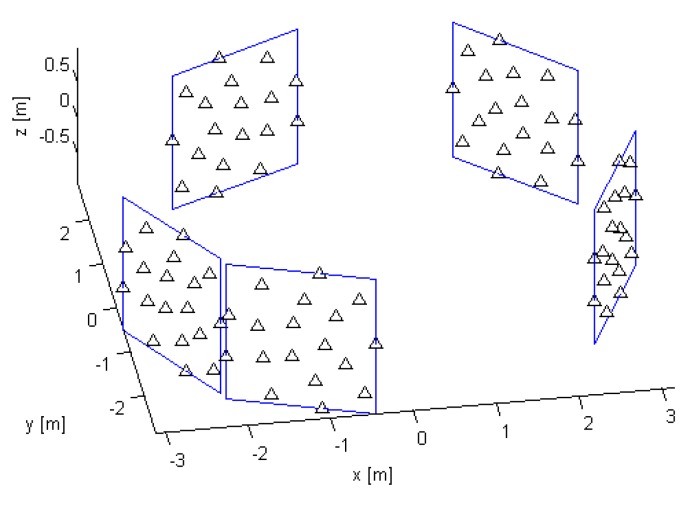
The Rx array geometry $G_{90}$.

As the previous results showed, $G_{5}$ suffered from the ambiguity problem (high side lobes), so we introduced a distributed massive-MIMO-like geometry, suited for installation and localization in a single room. Geometry $G_{90}$ is a 90-element array formed by replacing every antenna in $G_{5}$ with a subarray of an 18-element acoustic camera [30] geometry scaled by a factor of 17/3. Each subarray was rotated so that it was in a vertical plane, displayed by a rectangle in Figure 13, and its broadside direction pointed to the center of the total array. According to the system model, the signals from all the $G_{90}$ antennas were processed as if they were a single large array; however, grouping antennas into subarrays can simplify the mechanical design and the installation of the system. We give results for Tx at $T_{1}=\left(0,0,0\right)$ and different scenarios regarding the presence/absence of multipath and interfering signals. The parameters of the LoS component were B=100MHz, ${\mathrm{SNR}}_{0}=10\;\mathrm{dB}$, and N=64. The statistical results were averaged over 8192 simulation runs. All of the magnitudes in the figures showing the results are in (dB). Similarly to $G_{5}$, $SNR_{0}=10\;\mathrm{dB}$ means that the SNRs in the channels were around 1dB.

**Figure 14:**
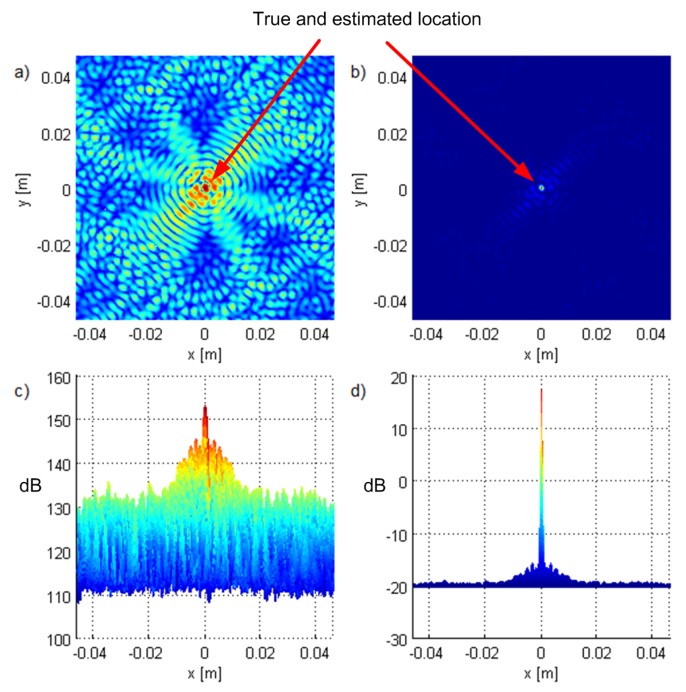
One-user LoS setting: top views of the criterion functions for $G_{90}$ and $T_{1}$ for (**a**) ML-US, (**b**) SCM-MUSIC, and the corresponding side views in (**c**,**d**) with the magnitude in dB.

Figure 14 shows the criterion functions in a single-user LoS-only scenario for both the ML-US and SCM-MUSIC algorithms. The ML-US side lobes were 8dB below the main lobe, whereas the SCM-MUSIC side lobes were 33dB below the main one. We used this scenario as a baseline to compare the results with multipath propagation and multiple transmitters against it.

### 4.4. Experiments with a Cluster of Scatterers and a Reflector

**Figure 15:**
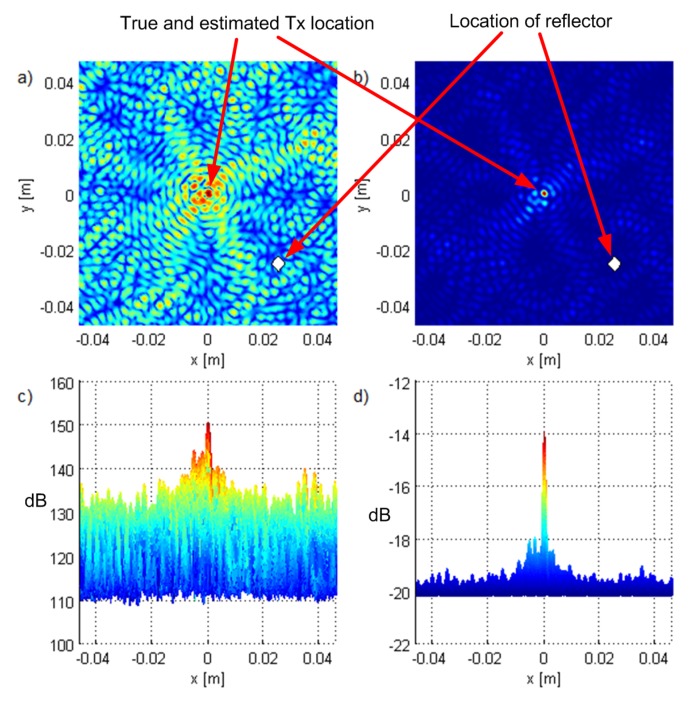
One user with a cluster of scatterers scenario: top views of the criterion functions for $G_{90}$ and $T_{1}$ for (**a**) ML-US, (**b**) SCM-MUSIC, and the corresponding side views in (**c**,**d**) with the magnitude in dB.

**Figure 16:**
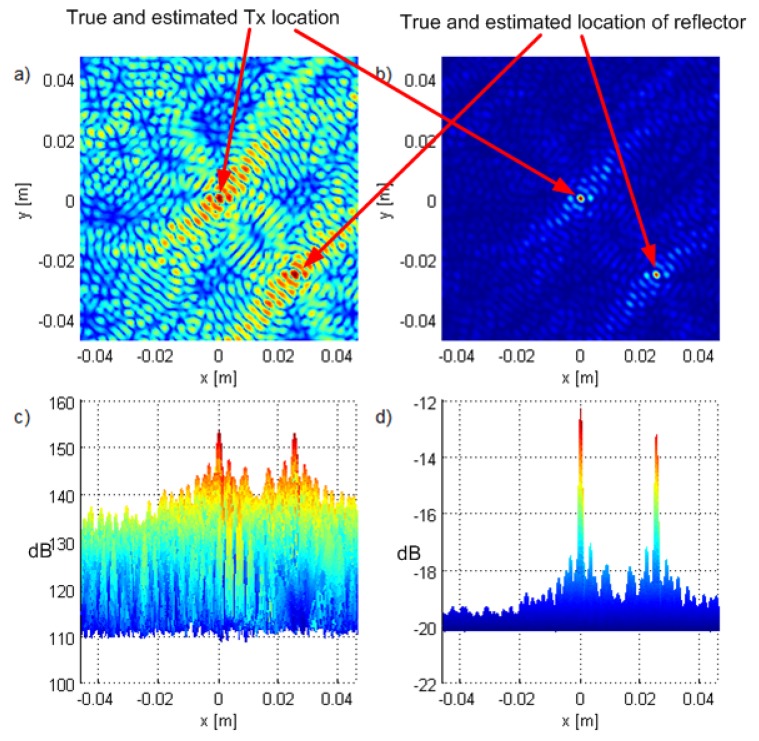
One user with an ideal reflector scenario: top views of the criterion functions for $G_{90}$ and $T_{1}$ for (**a**) ML-US, (**b**) SCM-MUSIC, and the corresponding side views in (**c**,**d**) with the magnitude in dB.

Figure 15 and Figure 16 show the results for scenarios with a small cluster of scatterers and an ideal reflector, respectively, positioned at $\left(0.025,-0.025,0\right)$ (denoted by a diamond). We modeled the cluster of scatterers as a point scatterer with randomized constant phase terms in the reflected (scattered) signal components. The power of the reflected signal components was pessimistically modeled to be inversely proportional to the squared sum of the distances the signal traveled before and after reflection. The only difference was that the carrier phases were modeled as random (uniformly distributed on $\left[0,2\pi \right]$) and deterministic, respectively. In the former scenario (Figure 15), the algorithms did not detect the reflector because the carrier phase of the reflected component was randomized. However, the waveform of the NLoS component was correlated with the useful (LoS) component, so the effect was an increase in side lobe magnitudes. Even though the shape of the criterion function was (qualitatively) similar to the one without multipath propagation as in Figure 14, the suppression of the side lobes was lower; it was roughly 6dB and 4dB for the ML-US and SCM-MUSIC algorithms respectively, instead of 8dB and 33dB, as in the LoS-only scenario in Figure 14. In the latter scenario (Figure 16), there was no notable increase in side lobes, but the reflector was detected as a separate source. SCM-MUSIC requires knowledge of the number of “sources”, so it is assumed known in the simulations. These setups represent extreme cases, and one may expect something in between in practice.

Table 2 shows the statistical performance of the algorithms for these experiments, but since we wanted to emphasize the effects of multipath components, we increased ${\mathrm{SNR}}_{0}$ to 20dB. The results are presented for different power ratios between the NLoS components and the LoS component, denoted $\delta_{\mathrm{NLoS}}$. For the sake of comparison, the results for the setting with only the LoS component are shown. The algorithms behaved similarly in these scenarios as their performance deteriorated due to multipath, but the RMSE was still well bellow $\lambda_{c}$. For NLoS levels of 10dB or more below the LoS component, which are realistic for the indoor environments in the mmWave, according to [11], the RMSE was by two orders of magnitude smaller than $\lambda_{c}$. This accuracy is important, because it enables the base station to focus energy to the Tx for down-link communication after the Tx is localized. Additionally, we generated the results for different scatterer/reflector positions inside the array (farther away from the Tx), as well as for N=512, but the results were only slightly different.

Table 2 shows that the localization methods proposed in the paper could achieve an accuracy much better than the carrier wavelength. On the other hand, the existing two-step localization methods, such as energy-based methods applied in mmWave massive MIMO systems, have drastically lower accuracies: the error is higher than the carrier wavelength, e.g., it is in the order of a meter [1,31] and 8mm,^32^]. However, the energy-based methods are easier to implement than coherent methods, especially in terms of synchronization (they can work with very rough synchronization).

### 4.5. Experiments with Two Transmitters

**Figure 17:**
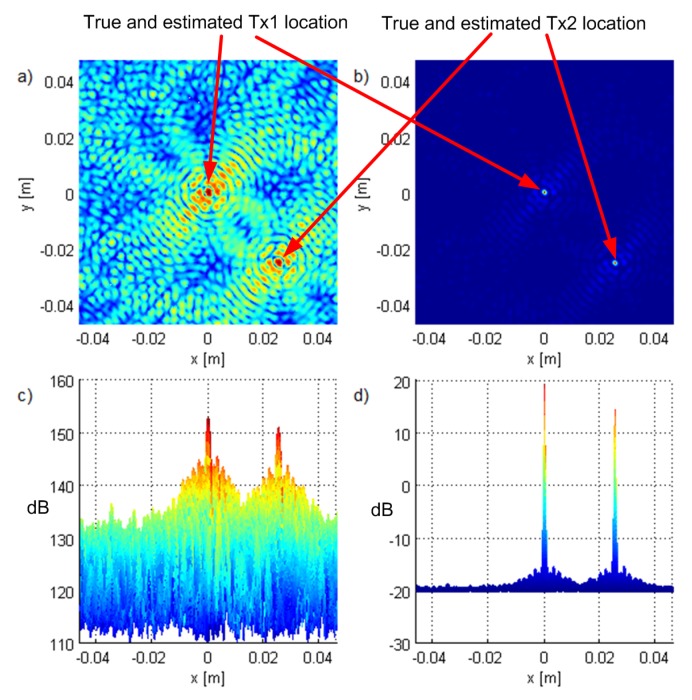
Experiments with two transmitters in LoS and with equal powers: top views of the criterion functions for $G_{90}$ and $T_{1}$ for (**a**) ML-US, (**b**) SCM-MUSIC, and the corresponding side views in (**c**,**d**) with the magnitude in dB.

**Figure 18:**
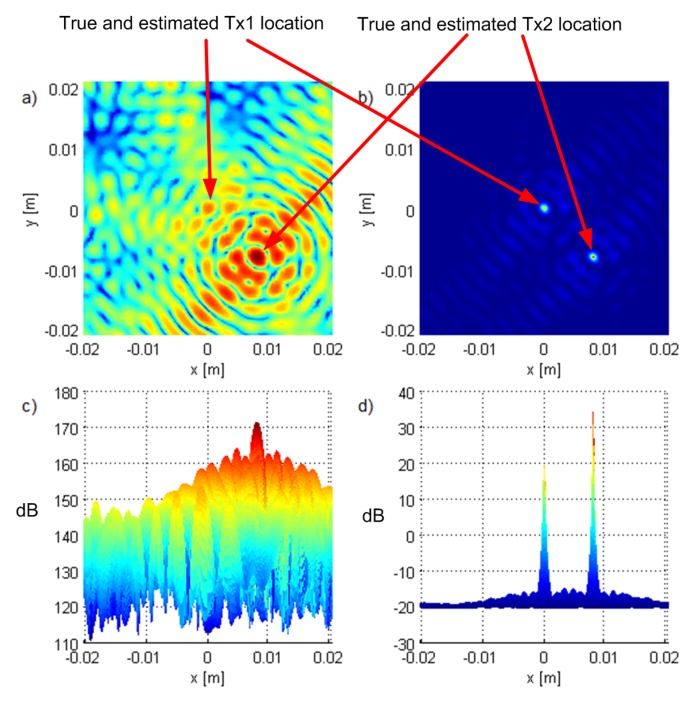
Experiments with two transmitters in LoS with a 10-dB difference in powers: top views of the criterion functions for $G_{90}$ and $T_{1}$ for (**a**) ML-US, (**b**) SCM-MUSIC, and the corresponding side views in (**c**,**d**) with the magnitude in dB.

We performed also experiments with two transmitters with equal transmit powers and located at (0.025,−0.025,0), thus separated by $7.07\lambda_{c}$. Figure 17 shows the results, and they suggest that both algorithms distinguish the transmitters. Note that the side lobe suppression is approximately the same as in the LoS-only scenario in Figure 14. In Figure 18, we plot the results of an experiment where the other user is at a (0.008,−0.008,0) ($2.26\lambda_{c}$ distance) and with a 10dB greater transmit power. ML-US could not estimate the position of the first Tx because its main lobe was lost in the side lobes corresponding to the interferer. SCM-MUSIC, however, localized the two sources successfully.

Table 3 shows the statistical performance of the algorithms when the interferer was located at (0.025,−0.025,0) for different power ratios between the interferer and the Tx, denoted $\delta_{\mathrm{IF}}$. We used ${\mathrm{SNR}}_{0}=20\;\mathrm{dB}$ for the same reason as in the multipath scenarios. For $\delta_{\mathrm{IF}}\leq 0\;\mathrm{dB}$, both algorithms performed well, with SCM-MUSIC performing almost as if the interfering signal were not present. For $\delta_{\mathrm{IF}}\geq 10\;\mathrm{dB}$, ML-US failed to localize the Tx (see Figure 18), whereas SCM-MUSIC performed well even when it was 30dB below the interferer. This is one advantage of SCM-MUSIC over ML-US, which justified its use despite the fact that ML-US was less complex. In other words, the SCM-MUSIC method was more robust in conditions with large differences in transmitted power between different transmitters.

We note one important consequence of the spatial selectivity of the methods discussed in the previous text. If a base station can use its distributed antenna array to distinguish between (and localize) two antennas of the same user terminal, separated by 5cm, then the base station can send two independent data streams toward these antennas, effectively increasing the throughput in the downlink toward that user in the same time-frequency resource.

**Figure 19:**
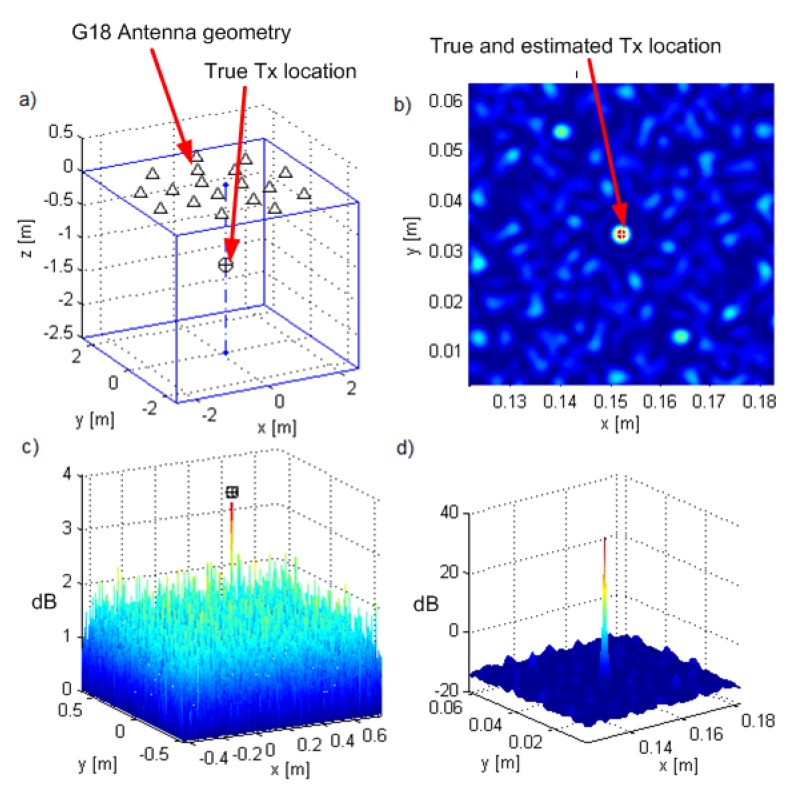
Results of localization for $G_{18}$: (**a**) Rx array geometry and Tx position, (**b**) ML-US criterion function over a smaller area, (**c**) ML-US over a larger area, (**d**) SCM-MUSIC over a smaller area with the magnitude in dB.

As an alternative to $G_{90}$, we introduced geometry $G_{18}$, which also suppresses side lobes, but is less expensive, both in terms of hardware and processing cost. Geometry $G_{18}$ is the acoustic camera geometry [30] scaled by a factor of 12, placed in the plane at z=0 (see Figure 19a). Figure 19b–d shows the results for $G_{18}$ and a source at $T_{3}=(0.152,0.033,$-1.2), which corresponds to a setup when the array is attached to the ceiling of a room and there is a transmitter 1.2m below. The ${\mathrm{SNR}}_{0}$ was set to 12dB, which entailed that the SNRs in the channels were between 4 and 10dB. The search grid was in the plane z=−1.2m, which contained the true location of the transmitter. The CRB for the Tx location was $2.93\times 10^{-9}\;m^{2}$, whose square root corresponded to $0.011\lambda_{c}$. The criterion function side lobes were suppressed (at least 43dB lower than the main lobe).

**Figure 20:**
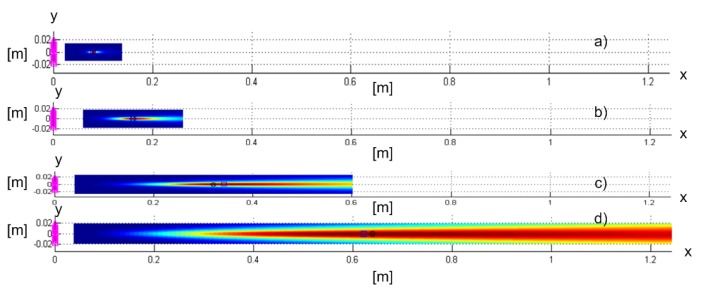
SCM-MUSIC criterion functions for localization of a transmitter at a distance of (**a**) 1, (**b**) 2, (**c**) 4, and (**d**) 8 apertures from the collocated massive antenna array.

The proposed methods were based on a model that treats the signal wavefront as spherical (they do not use the planar wave assumption). We now show that they were not only able to estimate the AoA of the received signal, but also to localize the transmitter using a collocated massive antenna array. We performed simulations for a uniform rectangular array with 16×16 antennas spaced $\lambda_{c}/2$ apart at $\nu_{c}=60\;\mathrm{GHz}$, centered at $\left(0,0,0\right)$ and lying in the yz-plane. The array aperture was then 7.5cm. We simulated a transmitter on the *x*-axis at a distance of 1, 2, 4, and 8 apertures away from the array. The criterion functions for these four Tx locations are shown in Figure 20a–d, respectively. The RMSE values along the *x*-axis for 8192 Monte Carlo runs for these locations were $0.048\lambda_{c}$, $0.18\lambda_{c}$, $0.7\lambda_{c}$, and $2.8\lambda_{c}$, respectively. The RMSE values along the *y*-axis were $0.0042\lambda_{c}$, $0.008\lambda_{c}$, $0.016\lambda_{c}$, and $0.032\lambda_{c}$, respectively. We concluded that the accuracy along the *y*-axis was better than the carrier wavelength, whereas along the *x*-axis, it was worse, but localization was still possible, thanks to the curvature of the wavefront. Furthermore, the RMSE along the *x*-axis increased with the distance from the array. To make the comparison fair, we chose different ${\mathrm{SNR}}_{0}$ for these Tx locations to make the actual SNRs in the receiving channels be around 4.5dB for each of the locations.

## 5. Conclusions

In this paper, we analyzed wideband direct localization of a transmitter in a multipath scenario with spatially-coherent LoS signal components. The signal model in this paper was both spatial-wideband and frequency-wideband, unlike most of the models in the existing literature. The same goes for the proposed localization algorithms. The simulation results for the proposed algorithms showed that they were statistically efficient. Further, they showed that the LoS-only localization RMSE could be two to three orders of magnitude lower than the carrier wavelength (even as accurate as one thousandth of the carrier wavelength) for a reasonable number of receiving antennas and system parameters, such as SNRs, the bandwidth, and the number of samples (such as 16 samples). The simulations also showed that localization RMSEs are two orders of magnitude lower than the carrier wavelength even in multipath and multiuser scenarios. Furthermore, relatively high accuracy can be achieved even for low SNR conditions and short observation intervals. Overall, the proposed methods were still numerically complex, and their further numerical optimization is a part of future research. The SCM-MUSIC algorithm had higher numerical complexity, but it was more robust in conditions with large differences in transmitted signal power from different sources; and it worked even in some cases when the ML-US algorithm did not. If a base station can localize two antennas in LoS conditions as separate sources, it can send two independent data streams toward them in the same time-frequency resource, while reducing interference to other points in the area. These results suggest that the performance (throughput, interference cancellation) of future wireless cellular systems, especially distributed massive MIMO, can dramatically improve. The presented results indicated that such systems have a great potential for location-aided communication, which is not fully recognized in the existing literature.

## Figures and Tables

**Table 1:** An example of the numerical complexity of the proposed algorithms.

Algorithm	Number of Multiplications	Number of Additions	Number of Exp Functions
ML-KS	$1.15\times 10^{9}$	$3.83\times 10^{8}$	320
ML-US	1280	640	320
SCM-MUSIC	1847	1192	320

**Table 2:** RMSEs in the presence of multipath propagation and ${\mathrm{SNR}}_{0}=20\;\mathrm{dB}$. IR, ideal reflector; CS, cluster of scatterers.

$\delta_{\mathrm{NLoS}}$	ML-US		SCM-MUSIC	
	IR	CS	IR	CS
0dB	$\frac{\lambda_{c}}{45.21}$	$\frac{\lambda_{c}}{40}$	$\frac{\lambda_{c}}{45.09}$	$\frac{\lambda_{c}}{39.67}$
−5dB	$\frac{\lambda_{c}}{76.29}$	$\frac{\lambda_{c}}{71.26}$	$\frac{\lambda_{c}}{75.95}$	$\frac{\lambda_{c}}{70.31}$
−10dB	$\frac{\lambda_{c}}{131.3}$	$\frac{\lambda_{c}}{126.3}$	$\frac{\lambda_{c}}{130.7}$	$\frac{\lambda_{c}}{124.1}$
−15dB	$\frac{\lambda_{c}}{225.2}$	$\frac{\lambda_{c}}{221.7}$	$\frac{\lambda_{c}}{223.8}$	$\frac{\lambda_{c}}{220.1}$
−20dB	$\frac{\lambda_{c}}{380.6}$	$\frac{\lambda_{c}}{371.7}$	$\frac{\lambda_{c}}{380.4}$	$\frac{\lambda_{c}}{375.9}$
LoS
only	$\frac{\lambda_{c}}{1090}$		$\frac{\lambda_{c}}{1070}$	

**Table 3:** RMSEs for scenarios with an interferer and ${\mathrm{SNR}}_{0}=20\;\mathrm{dB}$.

$\delta_{\mathrm{IF}}$	ML-US	SCM-MUSIC
+30dB	N/A	$\frac{\lambda_{c}}{604.6}$
+20dB	N/A	$\frac{\lambda_{c}}{1019}$
+10dB	N/A	$\frac{\lambda_{c}}{1061}$
0dB	$\frac{\lambda_{c}}{248.2}$	$\frac{\lambda_{c}}{1059}$
−10dB	$\frac{\lambda_{c}}{776.3}$	$\frac{\lambda_{c}}{1063}$
no interf.	$\frac{\lambda_{c}}{1090}$	$\frac{\lambda_{c}}{1070}$

## Abbreviations

The following abbreviations are used in this manuscript:

2G	second generation
3D	three-dimensional
5G	fifth-generation
A/D	analog-to-digital
AoA	angle of arrival
CRB	Cramér–Rao bound
D/A	digital-to-analog
DFT	discrete Fourier transform
DPD	direct position determination
FFT	fast Fourier transform
LAC	location-aided communication
LBS	location-based services
LoS	line-of-sight
MCME	maximum covariance matrix eigenvalue
MIMO	multiple-input multiple-output
ML	maximum likelihood
ML-KS	maximum likelihood known sequence
ML-US	maximum likelihood unknown sequence
mmWave	millimeter-wave
MSE	mean-squared error
MUSIC	multiple signal classification
NLoS	non-line-of-sight
RF	radio frequency
RMSE	root-mean-squared error
RSS	received signal strength
SCM-MUSIC	steered covariance matrix multiple signal classification
SNR	signal-to-noise ratio
TDoA	time difference of arrival
ToA	time of arrival

## Appendix A. Derivation of Maximum Likelihood Algorithms

If $Q_{m}=F^{H}D_{t_{0}+d_{m}/c}F$ and ${∥\cdot ∥}_{F}$ is the Frobenius norm, then the signal in channel *m* is given by $u_{m}=Q_{m}s+\eta_{m}$, and the log likelihood function (without the additive constant) multiplied by −1 becomes:

(A1) $$\begin{array}{cc} f_{\mathrm{LL}}= & \sum_{m=1}^{M}\frac{1}{\sigma_{m}^{2}}{∥u_{m}-Q_{m}s∥}_{F}^{2}\\ = & \sum_{m=1}^{M}\frac{1}{\sigma_{m}^{2}}\left(u_{m}^{H}-s^{H}Q_{m}^{H}\right)\left(u_{m}-Q_{m}s\right). \end{array}$$

According to the ML method, the estimate of the vector of unknowns, α, is the one that minimizes $f_{\mathrm{LL}}$. For convenience, define $\underline{u}={\left[u_{1}^{H}/\sigma_{1},u_{2}^{H}/\sigma_{2}\ldots u_{M}^{H}/\sigma_{M}\right]}^{H}\in C^{MN\times 1}$ and $\underline{Q}={\left[Q_{1}^{H}/\sigma_{1},Q_{2}^{H}/\sigma_{2}\ldots Q_{M}^{H}/\sigma_{M}\right]}^{H}\in C^{MN\times N}$.

ML-KS: If the sequence s is considered known, then the estimate of $\alpha =\left(t_{0},x,y,z\right)$ is the one that minimizes:

(A2) fKS1=fLL=∥u_−Q_s∥F2=u_Hu_−2Re(u_HQ_s)+ζsHs,

where ${\underline{Q}}^{H}\underline{Q}=\sum_{m=1}^{M}Q_{m}^{H}Q_{m}/\sigma_{m}^{2}=\sum_{m=1}^{M}Q_{m}^{-1}Q_{m}/\sigma_{m}^{2}=I_{N}\sum_{m=1}^{M}1/\sigma_{m}^{2}=\zeta I_{N}$. Since ζ, the first term, and the third term in (A2) are constants, minimizing *f*_KS1_ is equivalent to maximizing:

(A3) fKS2=Re(u_HQ_s)=Re∑m=1M1σm2umHQms

ML-US: If **s** is considered unknown, then the estimate of $\alpha =\left(x,y,z\right)$ is the one that minimizes:

(A4) $$f_{\mathrm{US}1}={∥\underline{u}-\underline{Q}\hat{s}∥}_{F}^{2},$$

where $\hat{s}$ is the value of s that minimizes *f*_LL_ for a given (*x, y, z*), or more formally $\hat{s}=\underset{s}{\arg\;\min\;}f_{\mathrm{LL}}$. The solution is:

(A5) $$\begin{array}{c} \hat{s}={\underline{Q}}^{†}\underline{u}={\left({\underline{Q}}^{H}\underline{Q}\right)}^{-1}{\underline{Q}}^{H}\underline{u}=\frac{1}{\zeta }{\underline{Q}}^{H}\underline{u}. \end{array}$$

The criterion function is then:

(A6) $$\begin{array}{cc} f_{\mathrm{US}1} & =\left({\underline{u}}^{H}-{\hat{s}}^{H}{\underline{Q}}^{H}\right)\left(\underline{u}-\underline{Q}\hat{s}\right)\\ \; & ={\underline{u}}^{H}\underline{u}-\frac{1}{\zeta }{\underline{u}}^{H}\underline{Q}{\underline{Q}}^{H}\underline{u}. \end{array}$$

The first term is a constant, so minimizing $f_{\mathrm{US}1}$ is equivalent to maximizing:

(A7) $$f_{\mathrm{US}2}={\underline{u}}^{H}\underline{Q}{\underline{Q}}^{H}\underline{u}.$$

Since ${\underline{Q}}^{H}\underline{u}=\sum_{m=1}^{M}\frac{1}{\sigma_{m}^{2}}Q_{m}^{H}u_{m}$, we have:

(A8) $$\begin{array}{cc} f_{\mathrm{US}2} & =\sum_{l=1}^{M}\sum_{m=1}^{M}\frac{1}{\sigma_{l}^{2}\sigma_{m}^{2}}u_{l}^{H}Q_{l}Q_{m}^{H}u_{m}\\ \; & =\sum_{l=1}^{M}\sum_{m=1}^{M}\frac{1}{\sigma_{l}^{2}\sigma_{m}^{2}}u_{l}^{H}F^{H}D_{d_{l}/c-d_{m}/c}Fu_{m}. \end{array}$$
